# Asperity level characterization of abrasive wear using atomic force microscopy

**DOI:** 10.1098/rspa.2021.0103

**Published:** 2021-06

**Authors:** Jack Walker, Jamal Umer, Mahdi Mohammadpour, Stephanos Theodossiades, Stephen R. Bewsher, Guenter Offner, Hemant Bansal, Michael Leighton, Michael Braunstingl, Heinz-Georg Flesch

**Affiliations:** ^1^ Wolfson School of Mechanical Engineering, Loughborough University, Loughborough LE11 3TU, UK; ^2^ Department of Mechanical Engineering, University of Engineering and Technology, Lahore 54890, Pakistan; ^3^ AVL List GmbH, Hans-List-Platz 1, 8020 Graz, Austria

**Keywords:** wear, abrasive, atomic force microscope, nanoscale, friction

## Abstract

Using an atomic force microscope, a nanoscale wear characterization method has been applied to a commercial steel substrate AISI 52100, a common bearing material. Two wear mechanisms were observed by the presented method: atom attrition and elastoplastic ploughing. It is shown that not only friction can be used to classify the difference between these two mechanisms, but also the ‘degree of wear’. Archard's Law of adhesion shows good conformity to experimental data at the nanoscale for the elastoplastic ploughing mechanism. However, there is a distinct discontinuity between the two identified mechanisms of wear and their relation to the load and the removed volume. The length-scale effect of the material's hardness property plays an integral role in the relationship between the ‘degree of wear’ and load. The transition between wear mechanisms is hardness-dependent, as below a load threshold limited plastic deformation in the form of pile up is exhibited. It is revealed that the presented method can be used as a rapid wear characterization technique, but additional work is necessary to project individual asperity interaction observations to macroscale contacts.

## 1. Introduction

Tribological contacts can be attributed to approximately 23% of global energy consumption, with 3% recognized as directly wear-related [1]. Holmberg & Erdemir [1] suggest the application of tribological research could reduce energy loss due to wear and friction, over the next 15 years, by 40%. To gain greater insight into the tribological phenomena of wear, experimental analysis at different length scales is necessary to develop knowledge that can be applied to multi-scale wear modelling. This study concentrates on wear observation at a nanoscale that is less understood in the literature in comparison with macroscale wear characterization (table 1).

**Table 1.** Nomenclature.

*A*	indentation contact area
*C_F_*	correlation factor
*C*_1,2,3_	geometry coefficient
E*	reduced elastic modulus
*F_f_*_[nN]_, *F_f_*_[V]_	friction force in Newtons/voltage
*h_c_*	plastic indentation depth
*H_l_*, *H*_0_	local hardness, bulk hardness
K*_a_*	Archard coefficient
*P*, *P*_max_	load, maximum load
*P_c_*	pull-off force
$\bar{P}$	ratio of applied load to adhesive forces
*R*	equivalent radius
*S_r_*	sliding distance
*V*	worn volume
*w*	work of adhesion
*z*_0_	equilibrium separation between atomic planes
Υ	elasticity parameter
*μ*	Tabor's elastic parameter
*μ*_fr_, *μ*_pl_, *μ*_adh_	coefficient friction/ploughing/adhesion
*χ*	JKR-to-DMT limiting factor

In this study, wear mechanisms of mechanical regimes are to be considered, of which there are many subdefined categories [2]. Surface failures due to sliding wear mechanisms that lead to plastic deformation [3], cracking and material removal are the focus of this study rather than fatigue. The ability to interpret between mechanisms for single asperity interactions is made using the difference in the volume of material displaced and that of the pile-up material as well as frictional characteristics.

Characterization of wear from a contact level using conventional tribometers, such as pin-on-disc, requires excessively prolonged experimental and measurement phases to form wear maps [4]. Wear on an individual asperity interaction basis has rarely been experimentally explored; with many of the original studies focusing on microscale, rather than on the nanoscale of asperities [4–6]. To characterize wear at the nano-length scale, one approach is to use an atomic force microscope (AFM) with an appropriately selected cantilever and tip. The use of such an instrument forms the basis of the methodology in the current study.

It is important to consider material physical properties at a nano-length scale as they are shown to be different to that of a bulk material. Nix & Goa [7] conducted an analysis on the indentation of crystalline materials to develop a bulk to nanoscale hardness connection. Bhushan & Nosonovsky [8] further developed this work and applied it to wear coefficients. The limitation of such relationships as indicated by Bushan & Nosonovsky is the lack of ability to measure the characteristic length scale, which enables the interpretation of nanoscale properties to the bulk material characteristic.

When observing contact mechanics at an asperity scale, adhesion is present between the two contiguous surfaces. Depending on the magnitude of the adhesion force of the contacting bodies, plastic deformation of soft materials may occur. Therefore, evaluation of the adhesion significance is imperative as part of an asperity level wear investigation, which can be conducted using Johnson & Greenwood's [9] adhesion map.

Many studies have conducted AFM wear mapping or characterization upon laboratory-scale single crystal silicon or using coating substrates such as diamond-like-coatings (DLC) [10–14]. Therefore, applying similar AFM wear characterization techniques to an engineering steel with grain boundaries will extend the applicability of the methodology to a wider range of commonly used materials. Furthermore, these studies limited their scope of application of AFM scratching to interfacial phenomena of micro-electromechanical systems, and nanomachining [11–14].

Hokkirigawa & Kato [5] experimentally explored different modes of abrasive wear using a micro pin-on-disc apparatus, with *in situ* scanning electron microscope (SEM) imaging. Wear mode frictional characteristics were observed, and three distinct responses were recorded. While the work shows a novelty in its *in situ* SEM imaging of wear generation at a microscale, it has limited applicability to real-world scenarios, where the wear particles are typically entrained into the contact normally exacerbating wear.

Celano *et al.* [14] conducted an AFM experiment using a diamond tip sliding in contact with silicon, silicon–germanium and germanium for the purpose of controlled material removal. Two contact regimes were reported with different wear rates. A load threshold was identified as the transition between ‘sliding’ wear and ‘sliding–ploughing’ wear; the latter included plastic deformation of the contacting area.

As described, AFM nanomachining or nano-scratching techniques in the literature have typically concentrated on silicon [14] and DLC coatings [8,10]. In this study, a scratch methodology using AFM has been developed to enable wear characterization of commercial multi-grain metallic substrates at nanoscale, which has not been reported hitherto, with the future intention of linking together with multi-scale wear phenomena. It is imperative to consistently induce wear through asperity level interaction for use as a building block for a multi-scale approach to wear modelling. Friction characterization of types of nanoscale wear is made possible through the use of AFM in lateral force mode (LFM). The effect of the length scale on the material properties is shown to play an integral role in the relationship between the degree of wear and load. The experimental data conform well to Archard's Law of adhesion at a nano-level for metallic substrates, where plastic deformation is dominant in the wear mechanism. The presented method will enable rapid and efficient wear characterization expandable across different scales of length.

## 2. Methodology

### (a) Analytical approach

The magnitude of wear in mechanical systems is normally calculated by relating contact conditions to the wear volume using linear proportionality. As per Holm–Archard's Law of adhesion [15,16], also used for abrasive contacts, a linear relationship was established between load *P*, bulk hardness of the softer body *H*_0_, sliding distance *S_r_* and the worn volume, *V*

2.1 $$V\propto \frac{PS_{r}}{H_{\text{0}}}.$$

The proportionality coefficient for the following relationship is the Archard coefficient, *K_a_*. It can be described as the latent attributes contributing to wear in the system that have not been directly observed, often including the material hardness [17]. A more traditional explanation is the probability of producing a wear particle. Archard's Law has been used on multiple scales from AFM [14] to tribometers [15] and component level such as gear pairs and bearings [18]. Justification for the use on the asperity scale comes from Holm's original work being based on the dislodging of individual atoms [16]. Molecular dynamic simulations between a diamond probe and a DLC coating has also found that Archard's model is applicable at the nanoscale [19]. Sha *et al.* concluded that the justifications for the applicability between scales are the true contact area being directionly proportional to the number of atoms in contact, which is in turn determined by load. Therefore, the mass transportation in the contact is proportional to the normal load and, while at a constant velocity, the distance slid [19]. In the current paper, this applicablity in an engineering material is experimentally investigated and shown, which is in line with the above-mentioned explanations and computational findings inthe literature.

The loads associated with wear are to be considered relative to the contact boundary conditions and the material properties of the surface. The hardness of the softer body is one of the predominant parameters determining the quantity of wear. During purely plastic deformation, Bowden & Tabor [20] reported that the contact mean pressure is equivalent to the hardness of the softer body. Local plastic onset is supported by the elastic deformation of material surrounding the contact site. Tabor [21] showed that this occurs when the Hertzian contact pressure approaches approximately 60% of its hardness. However, during a macroscale sliding contact, one will observe elastic as well as plastic deformation. In the absence of adhesion, and predominant elastic deformation, a contact area can be approximated by Hertzian contact analysis.

Friction is a consequence of contacting surfaces and has been shown to be proportional to the asperity contact area, rather than the nominal contact area [20]. Friction that occurs during dry contacts comprises adhesion, and ploughing in the absence of third-body abrasion

2.2 $$\mu_{\text{fr}}=\mu_{\text{pl}}+\mu_{\text{adh}}.$$

When mechanical wear occurs in a system, it is important to consider how this will affect the system's frictional characteristics and ultimately its efficiency. Not only will wear change the contact conditions in terms of real contact area but will introduce third-body particles. If the particles are harder, such as ferrous oxides, the substrate bodies may display greater wear. To account for them as a latent attribute, a reciprocating sliding motion with suitable sliding distance must be selected. This is observed in the friction time history, as well as the wear rate of the system.

The role of adhesion is assessed using Johnson & Greenwood's adhesion map [9]. To use the map, two parameters must be computed, *γ* and $\bar{P}$. *γ* is best described as a ratio of the elastic displacement during surface separation to the range limit of the surface adhesive forces (equation (2.3)). The significance of adhesion, in the contact, is then determined by the ratio, $\bar{P}$, between the normal load applied, *P*, and the adhesive separation force (*πwR*) as per equation (2.4)

2.3 γ=1.16 μ≡1.16(Rw2E* 2z03¯)1/133

and

2.4 $$\bar{P}=\frac{P}{\pi wR}.$$

Table 2 provides a summary of the characteristics each model represents and how their work of adhesion differs. The separation force, *P_c_*, can be calculated using AFM, and hence the work of adhesion computed. By identifying the appropriate contact model, the role of adhesion can be realized.

**Table 2.** Overview of contact continuum models.

adhesive model	characteristics	work of adhesion
Bradley	small rigid spheres	*w* = −*P_c_*/4*πR* [22]
	adhesion force significant relative to the applied load	
Derjagin–Muller–Toropov (DMT)	small stiff spheres	*w* = −*P_c_*/2*πR* [23]
	long-range adhesive forces activeoutside the contact area	
	adhesion force significant relative to the applied load	
Hertzian	adhesion forces are negligible, i.e. <5%	n.a.
Johnson–Kendall–Roberts (JKR)	large compliant spheres	*w* = −*P_c_*/(3/2*πR*) [24]
	short-range adhesive forces activewithin the contacting area	
	adhesion force significant relative to the applied load	
Maugis–Dugdale (MD)	transition between DMT and JKRcharacteristics	*w* = −*P_c_*/*χπR* [25]
	adhesion force significant relative to the applied load	where *χ* limits are defined by DMT and JKR

### (b) *In situ* wear and friction experiment using atomic force microscope

**Figure 1.**
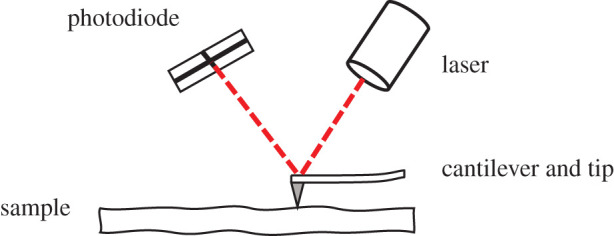
AFM set-up. (Online version in colour.)

AFM allows for *in situ* removal of material, as well as measurements of friction during LFM and topography in contact mode, with a vertical resolution of sub-nanometre (figure 1).

**Figure 2.**
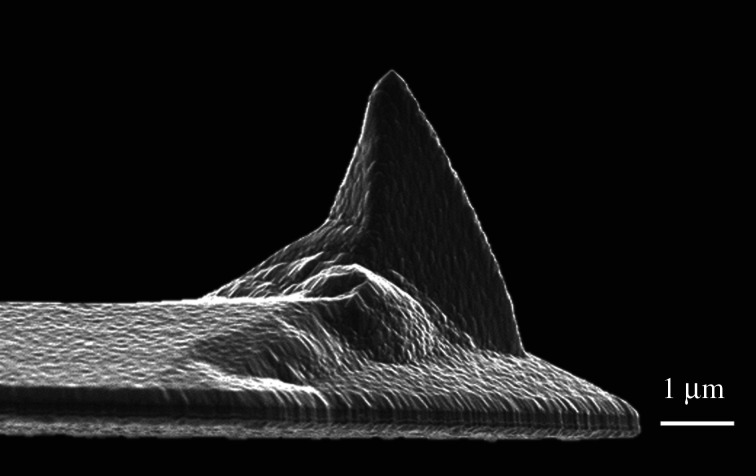
LEO 1530-VP SEM image of a new Adama NM-TC probe. (Online version in colour.)

The topography, friction and wear characteristics of surfaces were studied through use of a Veeco Dimension-3100 AFM. The friction and wear characteristics were determined operating the AFM in LFM [26–28]. In LFM, the probe scans in a direction normal to the cantilever beam using a trace minus retrace method. The characterization was conducted using a highly doped boron cantilever with a single diamond crystal tip, nominal cantilever stiffness 350 N m^−1^ and tip radius 90 nm. The AFM probe was selected specifically for its properties of high wear resistance and cantilever stiffness to induce wear of the substrate through plastic deformation of asperities in line with [29,30]. The scratches were completed with applied normal load varied from 7.5 to 157.3 µN and constant sliding velocities ranging from 1 to 10 µm s^−1^ on a polished mirror-finish substrate with RMS surface roughness of 4 nm. The tip radius was also measured experimentally using LEO 1530-VP high-resolution field emission SEM (figure 2). The effect of humidity on friction was not considered due to the relative insignificance of humidity frictional forces to the normal load applied. When humidity exceeds 65%, meniscus bridges can form over asperities increasing the coefficient of friction (CF). The adhesive forces were shown to be in the range of 0−200 nN [28].

The AFM probe cantilever was calibrated before each measurement with a blind calibration method [31,32], which required using a monolithic silicon nitride calibration sample with known frictional characteristics. A standard cleaning procedure, using methanol, was adopted to clean the calibration sample. For the calibration, a 1 µm^2^ area was scanned with a frequency of 2 Hz and sampling size of 256 × 256 data points. The voltage signal from the force transducer, which indicates the load, was converted into force units. To do this, a force–distance curve was produced while the AFM was operating in the ramping mode. The distance is multiplied by the cantilever stiffness to determine force. The sensitivity load–voltage slope is then multiplied by the applied voltage to determine the normal applied load, as explained in [28,31–33]. The calibration factor *C_F_* was then determined by measuring the friction force, by curve fitting for a range of applied loads, using the calibration sample and equation (2.5) in line with [28]

2.5 $$C_{F}=\frac{0.019\;F_{f[\text{V}]}}{P}.$$

The calibration factor represents, for a range of applied loads, the response of the AFM tip in use. During the wear test, the friction was then determined as

2.6 $$F_{f[\text{nN}]}=\frac{F_{f[\text{V}]}}{C_{F}}.$$

The wear characterization of the surface required an initial topography measurement at a low applied load. The AFM was then disengaged from topography mode and re-engaged in LFM. The topography was measured with the scanning speed of 10 µm s^−1^ in contact mode for a sample area of 10 × 10 µm^2^ with data array of 256 × 256 data points. The surface was then scratched, over the scan length of 5 µm by disabling the slow scan axis, for eight trace–retrace passes in LFM. Friction was measured while the scratch was produced. A probe scanning velocity of 1 µm s^−1^ was held constant. The tip was disengaged and re-engaged to measure the topography after the initial eight passes. The process was repeated five times to achieve a substantial wear depth for a total of 40 passes on a single line scan. A pass is defined as a single motion across the pre-defined scratch length in either direction, trace or retrace. To ensure repeatability, the overall process was repeated at four different locations on the surface. To avoid the effect of piezo drift between the scratch location relative to the tip with multiple re-engagements, the wear test was also conducted in a continuous manner with wear depth characterization being post-scratching at the conclusion of 40 passes. For repeatability purposes, this was completed for 10 repetitions. The repetitions were spaced 100 µm apart to ensure each individual scratch was not influenced by another. The current set-up does not include an *in situ* SEM imaging but has optical imaging capability. *In situ* SEM imaging may help to control the process better, as it develops.

### (c) Nanoindentation

When conducting studies of materials at asperity level, the bulk properties are not always applicable and localized properties are necessary to describe interactions, such as nanohardness. The nanohardness of the AFM test specimen used in this study was characterized using a nano indenter (Micro Materials NanoTest 600) with a Berkovich tip, 100−500 nm tip radius. Nine single-point indentations were performed per load ranging between 7 and 35 mN. The hardness was calculated as per Oliver & Pharr's methodology [34]

2.7 $$H_{l}=\frac{P_{\text{max}}}{A(h_{c})},$$

where *H_l_* is the nanohardness, *P*_max_ is the maximum load applied and *A* is the projected area of the contact, which is a function of the plastic depth *h_c_*. An area function for the Berkovich tip's projected area A was used in the following format:

2.8 $$A=\;C_{1}\;+\;C_{2}h_{c}+C_{3}{h_{c}}^{2},$$

where *C*_1−3_ are constants that describe the geometry of the indenter tip.

The measurements are subject to the ‘Indenter Size Effect’ (ISE). Milman *et al.* [35] demonstrated that the influence of the ISE explains that the decrease in indent area halts plastic deformation; plastic strain is somewhat reduced while the elastic strain increases. However, the summation of elastic and plastic strain is constant with indentation size. Plastic deformation is diminished by an increase in dislocation density, which serves to increase the local hardness of the material; therefore, at lower loads, the local hardness increases. The inclusion of the nanohardness is realized in the Archard coefficient for nanoscale computation of the wear volume.

## 3. Results and discussion

**Figure 3.**
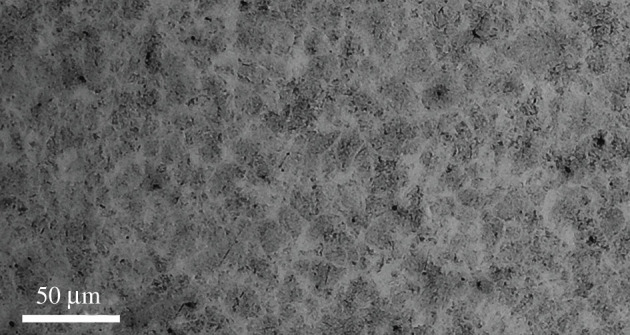
Microstructure of AISI 52100 surface etched with 2% Nital using an optical microscope.

A commercial steel, AISI 52100, was selected as the substrate for this study due to its application in many sliding contact conjunctions such as cam-tappets. The material was through hardened, water quenched from 805–820°C, tempered at 250°C and ultimately air cooled. The specimen was mirror-polished then etched using 2% Nitol to reveal the microstructure (figure 3). An optical microscope (Zeiss Primotech) with an objective lens of 20× was used to image the surface.

AISI 52100 steel is not a uniform structure and has a microstructure of tempered martensite with a body-centred tetragonal structure and 10–15% retained austenite with a face-centred cube structure. The grain structure is a homogeneous martensitic matrix with spheroidized (Fe,Cr)_3_C carbides. The material composition of AISI 52100 is presented in table 3.

**Table 3.** AISI 52100 material composition.

C	Mn	Si	Cr	Ni	Mo	Cu	S	P
0.95–1.10	0.20–0.50	<0.35	1.30–1.60	—	—	<0.025	<0.025	—

### (a) Nanohardness of substrate

**Figure 4.**
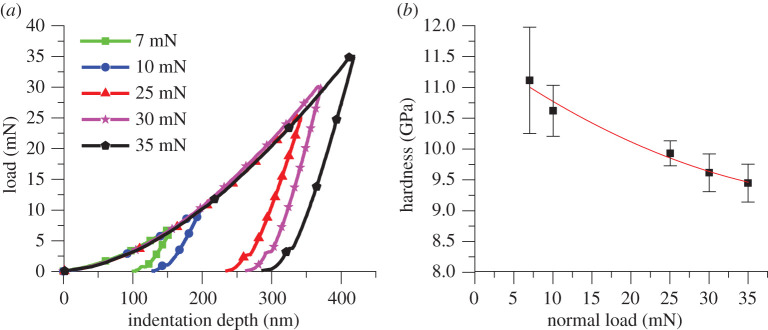
Average of nine nanoindentation repetitions at each load 7−35 mN. (*a*) Load–penetration graphs for nanohardness test and (*b*) hardness–load relationship. (Online version in colour.)

Rockwell *C* hardness tests of AISI 52100 indicate a hardness value of approximately 8.5 GPa [36], which can be considered as the bulk characteristic, but as is shown by the nanohardness tests conducted in this study, the hardness at a localized nanoscale increases with lower load. Measurements were performed by a Berkovich tip between loads of 7 and 35 mN with a fixed load and unload time period of 5 s starting from 0.03 mN to the given load. The indenter's contact velocity was 0.3 µm s^−1^ and a dwell period at the given maximum load of 5 s. The load penetration graphs are presented in figure 4 and show the nanohardness computed as per the Oliver & Pharr methodology [34] as highlighted in §2c for the investigated substrate.

The localized nanohardness is realized within the Archard coefficient; therefore, rearranging equation (2.1) and establishing the proportionality coefficient produces the following relationship between worn volume, load and sliding distance

3.1 $$V=K_{a}PS_{r}.$$

### (b) Degree of adhesion in the contact

**Figure 5.**
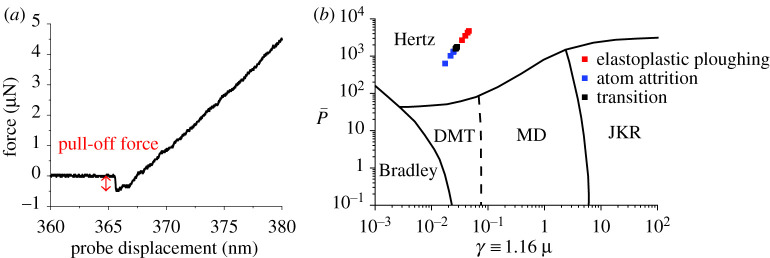
(*a*) AFM force–displacement curve for pull-off force determination. (*b*) AFM continuous reciprocating scratch plotted on adhesion map [9]. (Online version in colour.)

As previously stated, it is necessary to verify the adhesive forces that are associated with the contacts. This assumption has a central role in the applicability of Archard's Law in this investigation, as adhesion can affect plastic deformation within a contact. By using Johnson & Greenwood's [9] adhesion map with equations (2.3) and (2.4), the role of adhesion in the contacts was identified. The pull-off force is determined using the ramp mode during the blind calibration process. Figure 5*a* shows the force–displacement curve for the AFM tip, and the Bradley work of adhesion equation (table 2) was used initially and then the Derjagin–Muller–Toropov (DMT). The premise for selecting the Bradley model initially was due to the high hardness of the AFM tip and sample. Irrespective of the choice between either model, the contact was located on the adhesion map in figure 5*b* within the Hertzian model. The role of adhesion is shown to be insignificant in comparison with the applied load.

### (c) Applicability of Archard's Law and nonlinearity of wear rate

A highly doped boron cantilever with a nominal spring constant of 350 N m^−1^ and a single diamond crystal tip was used to perform the scratch and measurements. A SEM was used to image the AFM tip before and after the experiments were conducted to assess the tip condition. Moreover, SEM was also used to image the scratches as cross-validation for the width of the scratches and observe any material pile up.

**Figure 6.**
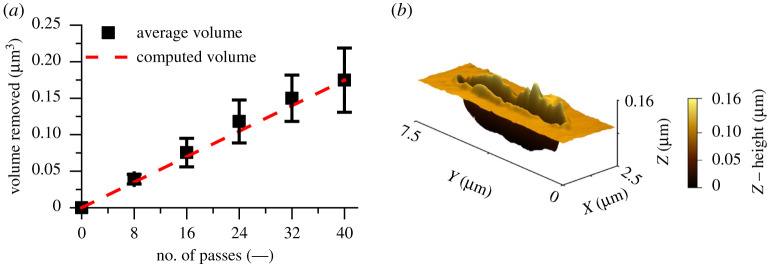
(*a*) AFM continuous reciprocating scratch average cumulative volume removed for a given number of passes. (*b*) A typical AFM topography measurement of a scratch after eight passes. Test parameters: load: 147.0 µN; sliding speed: 1 µm s^−1^; distance slid: 40 µm. (Online version in colour.)

The volume removed per eight passes using the continuous *in situ* reciprocating scratch and topography measurement methodology was calculated using a ‘topography differencing method’ [37]. Figure 6 shows a reciprocating scratch over 200 µm total sliding length at a sliding speed of 1 µm s^−1^ with an applied load of 147.0 µN. A typical AFM post-scratch topography measurement having completed 40 passes is displayed in figure 6*b*. A linear Archard wear coefficient was computed using the overall removed volume, and as shown in figure 6*a* showed generally good conformity to the experimental data for the given load. Despite applying a linear relationship, there is an inherent nonlinearity due to the evolving substrate's topography becoming conformal to the AFM tip. However, justification for applying a linear relationship is the fact that the maximum deviation between the average measured and computed volumes based on a linear relationship is 6.32%. Aghababaei *et al.* [6,38] have commented that the linearity of nanoscale wear is dependent upon the adhesion, material properties and surface roughness. The initial roughness of the substrate is 5 nm and is expected to have minimal influence in this AFM study. Moreover, as shown in the present research, adhesion does not play a significant role in the contact, especially at higher loads between the tip and the substrate, which justifies the insignificant effect of nonlinearity.

### (d) Interrupted versus continuous scratch

**Figure 7.**
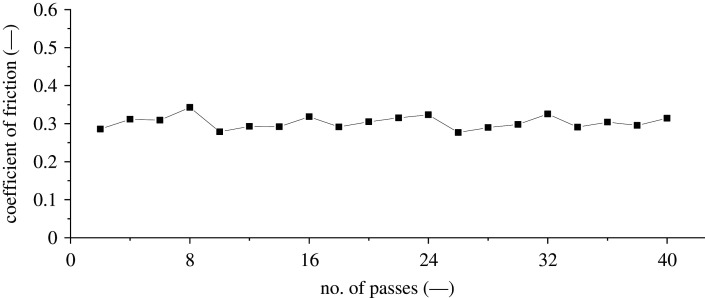
AFM interrupted reciprocating *in situ* scratch averaged trace–retrace coefficient of friction measurement versus number of passes. Test parameters: load: 147.0 µN; sliding speed: 1 µm s^−1^; distance slid: 200 µm.

During the implementation of an interrupted scratch-measurement technique, piezo drift of the AFM tip occurred, which may have influenced the friction and wear results. During this study, the interrupted methodology is defined as taking topography measurements every eight passes and then continuing the scratch process at the same site. The influence of continuous re-engagement of the AFM tip was evident in the friction results, as shown by the ‘ratcheting’ profile in figure 7. The re-engagement cyclic friction behaviour is due to a partially unploughed surface being contacted. Consequently, the re-engaged tip begins the continued scratch with a reduced friction force but gradually increasing with the number of passes before another topography measurement was taken.

**Figure 8.**
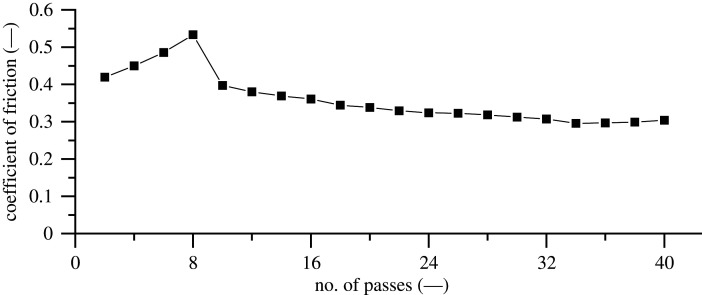
AFM continuous reciprocating scratch averaged trace–retrace friction measurement. Test parameters: load: 147.0 µN; sliding speed: 1 µm s^−1^; distance slid: 200 µm.

A reciprocating continuous scratch methodology with pre- and post-topography imaging was conducted to avoid the effects of piezo drift and that of entrained wear particles influencing an online scratch depth measurement. Figure 8 shows the variation of CF without disengagement of the AFM tip from the surface. Conditions are kept identical with those of figure 7 to compare the effect of interruption and drift in the measurement results. The first eight passes experienced an increase in contact area due to pile up and surface plastic deformation, corresponding to an increase in the CF. Figure 8 shows a greater initial coefficient of friction, which then reduces after the end of the first eight passes to a similar magnitude observed in figure 7. It is believed to be influenced by oxidation and the roughening of the surface in combination. A similar behaviour is observed by Bhushan in an AFM friction study of silicon [39]. Results also show that after reaching steady-state CF values, the results of interrupted and continuous experiments are in good agreement, revealing negligible effect of piezo drift.

### (e) Regimes of wear under different ranges of load

**Figure 9.**
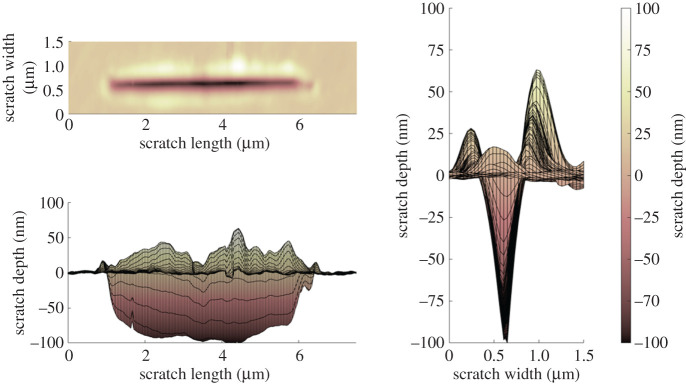
AFM continuous reciprocating scratch topography measurement. Test parameters: load: 147.0 µN; sliding speed: 1 µm s^−1^; distance slid: 40 µm. (Online version in colour.)

Figure 9 shows the topography measurement of a continuous scratch completed at a normal load of 147.0 µN. The groove volume is defined as the volume of material removed below the average surface plane height, while the pile-up volume is observed on the periphery of the groove sitting above the average plane height (0 nm; figure 9). Under high asperity load, it can be seen that the pile up to groove volume is significant, and a ductile response to the contact has been recorded. Figure 9 displays a wear scar with a groove volume of 0.089 µm^3^ in comparison with a pile-up volume of 0.048 µm^3^. The resulting groove geometry shows a high degree of conformity to the tip geometry; the grooves' ‘*V*’ angle is approximately 65°, while the tip angle for the given depth was approximately 60° as measured from SEM images. The difference is explained by the thermal drift of the AFM tip.

Despite the completion of 40 passes in the aforementioned interrupted and continuous methodologies (figures 7 and 8), steady-state friction was not achieved. Therefore, the complete sliding distance was increased from 200 to 10 000 µm; the scratch length remained 5 µm, but the number of passes increased to 2000. The extended sliding distance experiment reveals the capability of the presented approach in capturing different regimes of wear and identifying them via friction evaluation.

**Figure 10.**
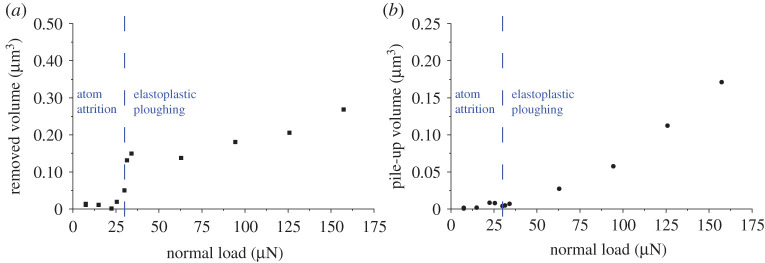
AFM continuous scratch volume measurement for the (*a*) removed volume and (*b*) pile up. Test parameters: load: 7.5–157.3 µN; sliding speed: 10 µm s^−1^; distance slid: 10 000 µm. (Online version in colour.)

Figure 10 displays the relationship between removed volume and pile-up volume to the applied normal load. Celano *et al*.'s [14] study of mesoscopic material removal of silicon substrates is analogous to the current experiment's metallic groove volume removal. Celano *et al.* referred to two different wear regimes, ‘sliding’ and ‘sliding–ploughing’. A more apt term of ‘sliding’ wear is an attrition of the substrate's atoms, while the ‘sliding–ploughing’ mechanism is predominately an elastoplastic deformation process. The atom attrition wear mode considering the local contact geometry is observed up to approximately 30 µN (figure 10). The transition between the mechanisms occurs at a load where the AFM tip penetrates the surface. A point contact maximum shear stress [40] of 12.2 GPa occurs at 30 µN, which is close to the theoretical shear strength of the material, 12.7 GPa. As with the ISE, as the indenter's radius decreases, the shear stress of the contact increases and the strength of the substrate approaches its theoretical shear strength. The theoretical shear strength of a crystalline metal is approximately equivalent to the shear modulus, 80 GPa for AISI 52100, divided by 2*π* [41]. Similar analysis was conducted by He *et al.* [42] and Morales-Rivas *et al.* [43], showing similar orders of magnitude for the shear stress at this scale for various steels. This correlation can potentially provide an opportunity to characterize the transition threshold based on the theoretical shear strength. Once the theoretical shear strength has been exceeded, a transition to the elastoplastic mode is evident. If hardness is to be defined as the resistance to plastic deformation and is mainly dependent on dislocation mobility, then at the nano-length scale, the shear modulus is a good indication of the scratch hardness of the material [44].

Atom attrition wear is observed when the removal of material from the surface occurs with little to no pile up. The elastoplastic mode combines material removal with significant pile-up volumes. Since the contact geometry of the surface changes with each pass, the contact area is increasing, and the dislocations have more free pathways. This likely explains the pile-up volume increasing with given load. The nonlinearity in the relationship between groove volume and normal load applied is not accounted for in Archard's Law of adhesion in its current form. There is a distinct step change in the ‘Archard coefficient’, the probability of wear particle production, where the threshold for metallic substrates begins to plastically deform.

**Figure 11.**
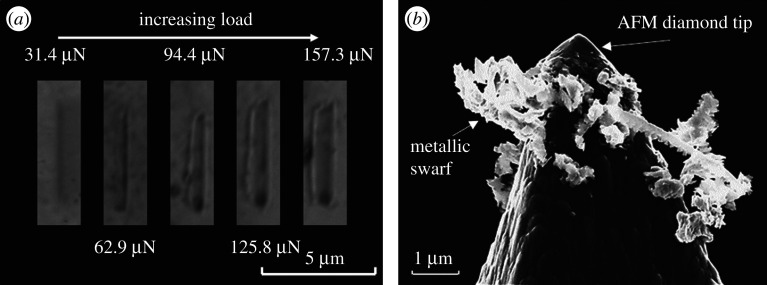
(*a*) SEM image of AISI 52100 substrate scratches. (*b*) SEM image of AFM tip post-scratch.

Figure 11*a* shows SEM images of scratches increasing in load from 31.4 to 157.3 µN, which clearly reveals the observed behaviour of increasing pile-up volumes as indicated by figure 10's AFM topography measurements. Moreover, the width of the scratches can be seen to increase giving an indication of depth. The elastoplastic mechanism is abrasive due to the surface penetration of the AFM tip and the ploughing of the surface that ensues. Figure 11*b* shows evidence of micro-metallic swarf from the steel surface adhered to the tip. This indicates an abrasive ploughing mechanism, which would be the most significant contributing mechanism to the overall removed volumes displayed in figure 10 after the critical load. The SEM images in figure 11*b* correlate well to a cutting abrasive wear mode classified by Hokkirigawa & Kato [5] in a microscale scratch study with *in situ* SEM.

During attrition wear, the AFM tip does not penetrate the surface and is found not to obey Archard's Law [45]. Wear under this regime may occur due to the breaking of chemical bonds subsurface via the scanning AFM tip at a constant load. It is thought that the tip initiates defects in the substrate that enables the breaking of bonds [46].

It is important to consider the wear of the tip since this will ultimately vary the contact geometry. SEM was used to image the AFM tip before and after 10 000 traces as well as the worn surfaces (figures 2 and 11*a*,*b*). The SEM images revealed no wear upon the diamond tip or alteration to the tip radius.

**Figure 12.**
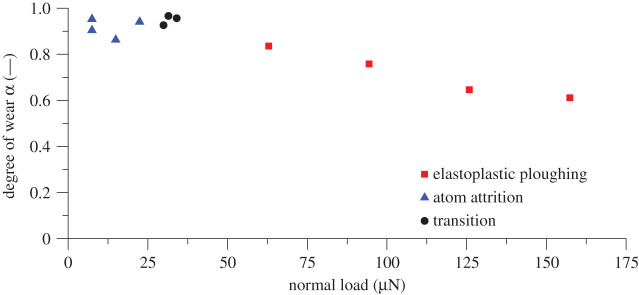
AFM scratch—degree of wear with respect to normal applied load. Test parameters: load: 7.5–157.3 µN; sliding speed: 10 µm s^−1^; distance slid: 10 000 µm. (Online version in colour.)

Hokkirigawa & Kato coined the term ‘Degree of Wear’ (DW). This is defined as the ratio of the actual wear volume to apparent groove volume [5]. Actual wear volume is defined here as the removal of material from the control volume, the control volume being the site of the scratch and near the vicinity of the scratch periphery. On inspection of the relationship between the DW and the normal load applied to the surface, it was found that the DW was decreasing with respect to increasing the contact pressure above the threshold of the wear mechanism transition (figure 12). This correlates to the decrease in nanohardness of the substrate with respect to increasing load (figure 3). This equally corresponds to Celano *et al*.'s commentary of a brittle-to-ductile transition of the substrate material [14].

**Figure 13.**
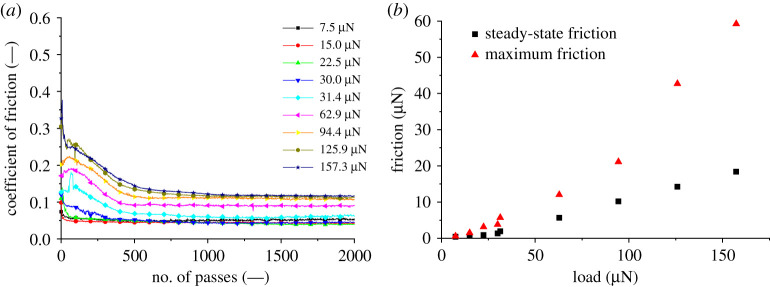
(*a*) AFM continuous reciprocating scratch friction measurement versus number of passes. (*b*) AFM continuous reciprocating scratch friction versus load. Test parameters: load: 7.5–157.3 µN; sliding speed: 10 µm s^−1^; distance slid: 10 000 µm. (Online version in colour.)

Figure 13*a* displays the CF against the number of passes of the tip completed with variation of the normal applied load between 7.5 and 157.3 µN over 10 000 µm. As previously mentioned, the DW and removed volume can be used to classify the wear that is occurring in the system. When the friction time history with varied loads are cross-correlated to the aforementioned measured values, a clear distinction can be made between the two wear mechanisms. The atom attrition mode has little variation of friction across the sliding distance, apart from the initial interaction with the surface, which could be attributed to oxides present on the surface. The elastoplastic ploughing mode shows an initial increase to a maximum value before steadily reducing to a steady state between 500 and 1000 passes. Figure 13*b* displays the relationship of steady state and maximum friction with load. The linear proportionality between normal load and steady-state friction can be considered as the shear strength of the nano-asperities. Above the load mechanism threshold, steady-state friction is only achieved once the contact area has significantly increased to support the higher loads. A similar concept to this is shakedown [47], where initially plastic flow occurs during an asperity contact but then the substrate is eventually capable of supporting the load elastically after several traverses of the point contact.

## 4. Conclusion

This study has investigated wear induced by a nanoscale asperity level contact between an AFM tip and a steel substrate, AISI 52100. Two types of wear mode, atom attrition and elastoplastic ploughing, were observed and seen to be load-dependent. Experimental data from an AFM *in situ* scratch-measurement technique showed good conformity to Archard's Law of adhesion at the nanoscale for the elastoplastic wear mode. Furthermore, AFM has been proven to have capabilities for rapid wear characterization at a nano-length scale. Once a relation is established between multiple length scales, which does not require multi-scale experimental wear characterization, AFM will offer the benefit of timely characterization against that of traditional macroscale tribometers. This is achieved through the ability of AFM to facilitate atomic attrition and elastoplastic forms of wear with accurate *in situ* topography measurements.

On a nanoscale, it was found that the apparent groove volume resulting from atom attrition and elastoplastic wear modes do not share a linear relationship across the range of normal loads applied. In fact for the steel substrate AISI 52100, a transition in wear behaviour was observed with respect to a load threshold for the given contact geometry, a similar behaviour which was observed by Celano *et al.* [14] in silicon substrates*.* However, it is proposed that the indentation size effect plays an important role in the wear mode transition. The behaviour observed below a critical load demonstrated a more brittle type of wear, since the load criterion was below that of an elastoplastic response, in line with greater surface hardness.

An elastoplastic mode was observed, which showed a more ductile behaviour, as exhibited from significant pile-up volumes. As the contact load increased the degree of wear reduced, which corresponded to more substantial plastic deformation in the form of greater groove depth and pile up.

Moreover, the variation of the coefficient of friction was observed with respect to reciprocating sliding distance and applied normal load over the scratch tests. Frictional characterization of the wear modes shows that a steady-state coefficient of friction is achieved by the atom attrition wear mode almost instantaneously in comparison with elastoplastic wear behaviour. The elastoplastic mechanism's coefficient of friction increases with growing real contact area, pile up and generation of wear debris, which become entrained in the contact. Ultimately, a steady-state friction is then observed, once the real contact area has grown to support the higher load without plastic deformation being predominate.

In order to take full advantage of the rapid wear characterization capabilities demonstrated using AFM, future work is required to superimpose the observations made of individual asperity interactions to that of a macroscale contact. Such an approach would benefit the design of mechanical sliding components such as gears for durability, efficiency, noise, vibration and harshness. It also suggested that AFM wear characterization can be expanded to a wider range of tip geometries and substrates to bring further clarity regarding the transition between the two wear regimes.

## Data accessibility

Data are available from the Dryad Digital Repository: https://doi.org/10.5061/dryad.s7h44j16j [48].
